# The impact of sleeve gastrectomy on pulmonary function tests and physical activity one-year after surgery

**DOI:** 10.1186/s12893-025-02804-0

**Published:** 2025-02-28

**Authors:** Mohammadmahdi Abbasi, Narjes Mohammadzadeh, Mohammad Hossein Pourgharib Shahi, AhmadReza Soroush, Reza Eslamian, Ali Mir, Fezzeh Elyasinia, Mohammad Talebpour, Khosrow Najjari, Hossein Zabihi Mahmoudabadi, Seyed Morteza Pourfaraji

**Affiliations:** 1 Department of Surgery, Shariati Hospital, Tehran University of Medical Sciences, N Kargar, Tehran, P9CP+JP2 Tehran Province Iran; 2 Department of Sports Medicine, School of Medicine Sports Medicine Research Center, Shariati Hospital Tehran University of Medical Sciences, Tehran, Iran; 3 Department of Surgery, Imam Khomeini Hospital, Tehran University of Medical Sciences, Tehran, Iran; 4 Department of Surgery, Sina Hospital, Tehran University of Medical Sciences, Tehran, Iran

## Abstract

**Introduction:**

Obesity can adversely impact respiratory function and limit physical activity (PA). Sleeve gastrectomy (SG) is an essential and uptrend treatment option for weight loss. However, the effect of SG on pulmonary function and PA in patients with obesity is still debated.

**Method:**

This is an observational study of 32 cases with obesity (BMI 43.86 ± 4.39) who underwent SG in a single center. Spirometry was performed before and 12 months after SG to investigate the pulmonary function of individuals. The main variables were forced expiratory volume (1s) (FEV1), Forced vital capacity (FVC), FEV1/FVC ratio, and maximum inspiratory pressure (MIP). The correlation of weight loss variables with findings was evaluated.

**Result:**

One year after surgery, patients lost an average of 23.42 kg (*P* <.001). The FEV1 and FVC were increased by 0.22 ml and 0.38 ml, respectively (*p* <.001). The absolute changes in FEV1 and FVS were significantly correlated with Total weight loss percentage (TWL). The 6-minute walking test (6MWT) results were significantly increased after surgery by 53.71 m (*p* <.001), and changes were correlated with TWL.

**Conclusion:**

More than significant weight loss, the SG can also significantly improve the respiratory function and PA of individuals with obesity 12 months after surgery. Additionally, there was a positive correlation between weight loss and modification in lung function tests. The findings required studies with larger sample sizes and longer follow-up times to confirm and clarify.

## Introduction

The rising prevalence of obesity in the last decades has led to a global concern around obesity and its associated medical complications. Researchers estimate the rise in obesity will almost double by 2025 globally, compared to 2010’s levels [1]. Obesity was also found to be a primary cause of years of life loss and disability-adjusted life years (DALYs) worldwide [2]. The literature revealed that bariatric surgery is a more effective intervention for managing severe obesity compared to medical therapy [3]. The rate of bariatric surgery gradually increased due to its advantages and very low mortality and morbidity rates. Among the various types of BS, sleeve gastrectomy has become more prevalent in recent years and is now considered one of the most performed bariatric procedures [4]. Despite the vast studies evaluating the superiority of BS over medical interventions regarding weight loss, managing glycemic indices, and reducing cardiovascular mortality, some other crucial aspects of these surgeries still need to be studied [3].

In some articles, the respiratory function was observed to be altered by weight gain through lung mechanical restrictions, diminished chest wall compliance, and increased inflammatory processes [5]. While moderate obesity seems to change the respiratory indices within the normal range, some studies found severe obesity could alter the total lung capacity (TLC), residual volume (RV), functional residual capacity (FRC), forced vital capacity (FVC), and forced expiratory volume in 1s (FEV1) [6]. A limited number of studies investigated the role of bariatric surgery on respiratory function. However, their findings indicated the benefit of bariatric procedures on lung function [7–9].

Despite the effective role of bariatric surgery in short-term total weight loss (TWL) and improving obesity comorbidity, the inevitable loss of lean body mass (LBM) raised concern for the long-term outcomes of bariatric surgery. The proportion of LBM loss to TWL ranged from 17.5 to 31.3% in various types of bariatric surgery [10]. Increased LBM may lead to unfavorable outcomes, including increased body weight, glycemic dysregulation, higher risk of bone fractures, decreased functional capacity, and reduced quality of life [11, 12]. Furthermore, several studies suggested excessive loss of LBM could negatively impact the gait speed, endurance, and ability to perform daily tasks [11–13]. Several mechanisms were suggested to explain the associations between weight loss and LBM. Vaurs et al. revealed higher levels of Thyroid-stimulating hormone (TSH) in patients with severe obesity, which significantly increased LBM loss. Post-surgical weight loss has been shown to reduce TSH levels in several studies, which could help preserve the LBM [12, 14, 15]. LBM plays a critical role in determining basal metabolic rate (BMR). A reduction in skeletal muscle mass (the primary component of LBM) after surgery can lead to a decline in BMR and lower energy expenditure during physical activity, potentially lowering long-term weight loss. Therefore, preserving LBM is essential for optimizing metabolic health and sustaining weight loss outcomes in individuals who undergo bariatric surgery [16]. Since physical activity (PA) is crucial in preserving LBM, assessing postoperative changes in PA and potential associated factors such as respiratory function, body composition, psychological status, and functional capacity is essential. This evaluation provides valuable insights into the long-term effects of BS.

Our primary aim in the current study was to investigate the one-year impact of laparoscopic sleeve gastrectomy (LSG) on respiratory function and functional capacity. Furthermore, the secondary goal was to assess the changes in TWL, EWL, Fat mass, and LBM of cases with obesity who underwent LSG.

## Materials and methods

In this observational cohort study, we investigated the patients who underwent LSG surgery and met the following criteria: (1) obesity class III or class II with at least one obesity comorbidity, (2) aged between 18 and 60 years, (3) having the ability to walk, (4) complete follow-up until 12 months after surgery. We also exclude cases with any of the subsequent statuses: (1) smoking habitus, (2) chronic lung diseases (asthma, COPD, obstructive sleep apnea, atelectasis), (3) cardiovascular disease (hypertension, history of MI or angioplasty), (4) prior history of abdominal surgery, (5) severe postoperative adverse event (e.g., gastrointestinal (GI) bleeding, GI obstruction), (6) major orthopedic disabilities, (7) lost to follow-up. All included patients underwent LSG surgery at Shariati Hospital (Tehran, Iran) between March 2023 and March 2024. After obtaining written informed consent, the required assessments were conducted at intervals of one month before and 12 months after the operation.

### Post-operative care

All subjects received standardized protocols after surgery, including nutritional counseling and physical activity recommendations. The nutritional program emphasized a low-calorie and high-protein diet. Two follow-up visits with dietitians 6 and 12 months after surgery were scheduled to evaluate patients’ adherence to recommendations. For physical activity, patients were encouraged to engage in moderate-intensity exercise, such as walking, for at least 150 min per week, consistent with global PA recommendations.

### Assessment of respiratory muscle strength

Respiratory muscle strength was evaluated using a properly calibrated digital mouth pressure meter of the Powerbreathe^®^ brand to measure the maximal inspiratory pressure (MIP), with a range of ± 300 cm H_2_O. The measurements were performed with the patients in sitting and upright positions after complete inhalation and exhalation. Each patient performed at least three maximal inspiratory efforts, with a 1-minute rest period between attempts to prevent fatigue. The highest value obtained from these efforts was recorded as the MIP for each patient. All assessments were conducted by trained personnel to maintain standardization across measurements. Subjects were instructed to refrain from caffeine consumption, strenuous physical activity, and heavy meals for at least 2 h before testing to minimize potential confounding effects on respiratory performance. The assessments were performed according to the American Thoracic Society/European Respiratory Society guidelines [17].

### Spirometry

Standardized resting spirometry was executed in a sitting position using a Spirobank II device (MIR, Rome, Italy). The values for forced vital capacity (FVC), expiratory flow in the first second (FEV1), and FEV1/FVC ratio were calculated using the previous method explained by Sahebi et al. in the Iranian population [18].

### Body mass composition

A body impedance analyzer (InBody 270, Biospace America, Inc.) was used to measure the body composition variables, including lean body mass, fat mass (FFM), and weight.

### Walking capacity

A 6-Minute Walk Test (6MWT) was accomplished to evaluate walking capacity. Previous studies have indicated that the 6MWT is a powerful test for assessing the functional capacity of patients with obesity [19]. Participants were instructed to walk for six minutes continuously at their own pace in a 30-meter-long hallway to measure their walking endurance.

### Physical activity

The physical activity of cases was investigated before and one year after surgery using the Global Physical Activity Questionnaire (GPAQ) [20]. This questionnaire has four sections evaluating activity at work, travel to and from places, recreational activity, and sedentary activities. The weekly minutes of activity were converted to metabolic equivalent (MET) based on the GPAQ guidelines [20]. Participants were categorized into three groups based on their weekly MET-minutes of physical activity (PA), as follows:

- High activity: Individuals who engaged in at least 7 days of walking, moderate-intensity, or vigorous-intensity activities, accumulating a minimum of 3000 MET-minutes/week.
- Moderate activity: Individuals who engaged in at least 5 days of walking, moderate-intensity, or vigorous-intensity activities, accumulating a minimum of 1500 MET-minutes/week.
- Low activity: Individuals who did not meet the high or moderate activity levels criteria.

This classification aligns with established methodologies used in previous studies to standardize PA levels and facilitate comparisons across populations.

The detailed information on classification previously mentioned in Boddu’s study [21].

### Statistical analysis

Data are presented in numbers and percentages for qualitative variables and mean ± standard deviation for continuous values. The distribution of data was checked using the Shapiro-Wilk test. In the next step, the differences between pre- and post-surgery values were evaluated using a paired T-test for variables with a normal distribution and a Wilcoxon-signed-rank test for other variables. The correlations between the anthropometric values change, and respiratory and PA parameters were assessed using the Pearson or Spearman correlation tests based on the distribution of variables. All statistical analyses were performed by IBM SPSS statistics (Version 26.0 for Windows, SPSS Inc., Chicago, IL). The reported *P* values were two-sided, and *p* values < 0.05 were considered significant in the current study.

## Results

The mean age at the time of surgery for 32 included patients was 42.91 ± 12.69 years, and patients were primarily women (*n* = 28). The secondary evaluation was performed 11.22 ± 0.79 months after surgery. The preoperative mean weight and BMI were 111.47 ± 13.98 kg and 43.86 ± 4.38 kg/m2, respectively. Following SG, the average weight loss was 23.42 ± 11.78 kg, and the mean BMI reduction was 9.14 ± 4.49 kg/m2. Additionally, patients lost 16.18 ± 8.44 kg fat mass and 7.26 ± 4.09 kg lean body mass after SG. The percentage of TWL and EWL were 20.64% ± 9.39% and 39.53% ± 18.295, respectively. All weight and body composition changes were significant (*P* value < 0.001) (Table 1).

**Fig. 1:**
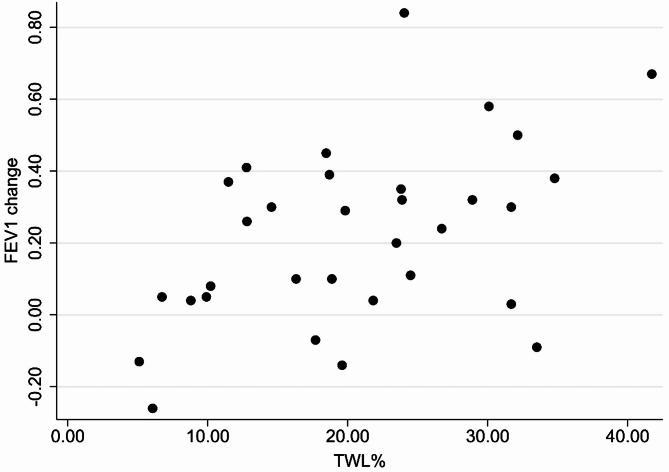
FEV1 change correlated positively with TWL% in patients with obesity who underwent sleeve gastrectomy (*r* =.470, *p* value = 0.007)

**Fig. 2:**
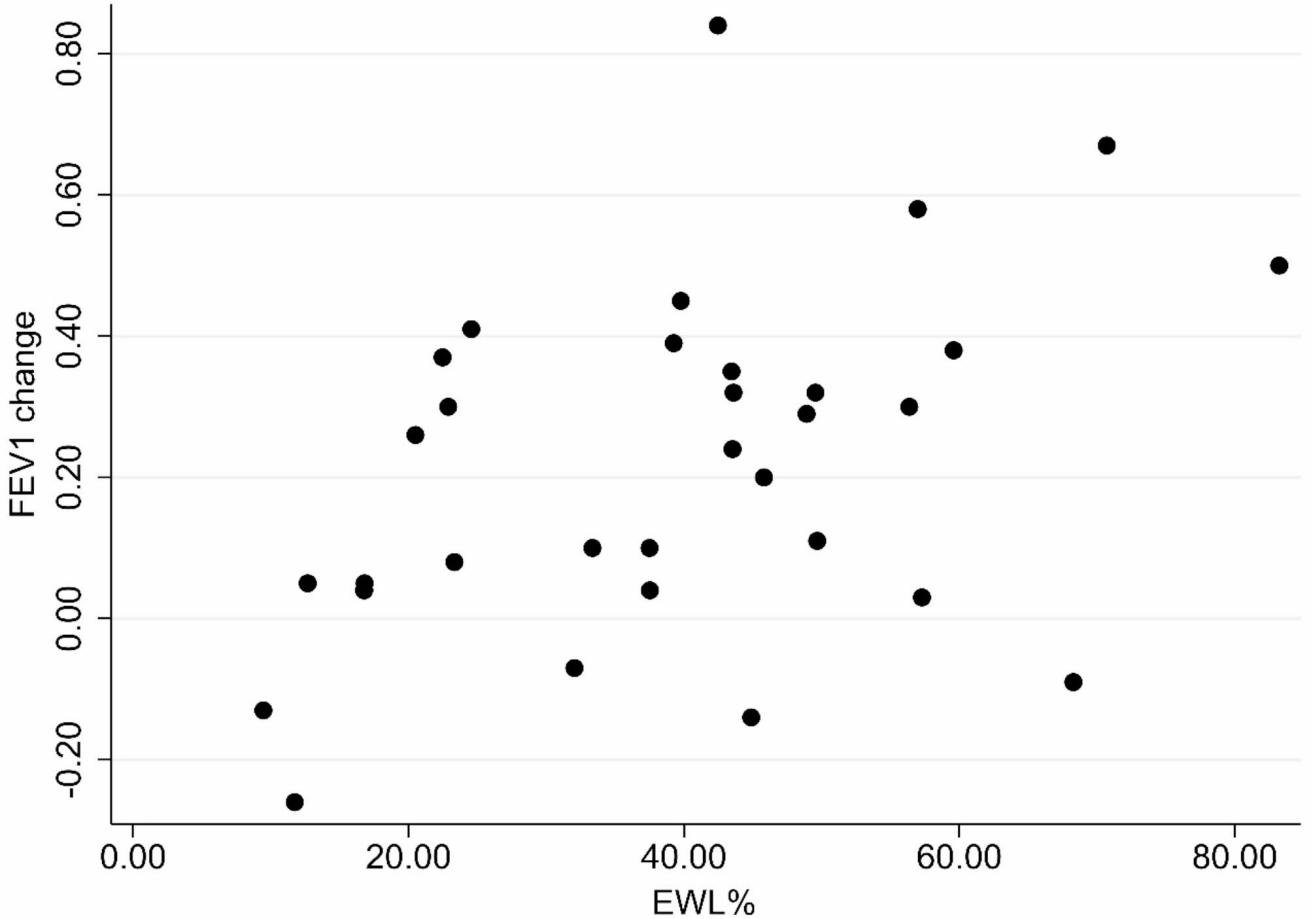
FEV1 change correlated positively with EWL% in patients with obesity who underwent sleeve gastrectomy (*r* =.433, *p* value = 0.013)

The mean MIP postoperative increase was 7.24 ± 17.42 cm H_2_O (*P* value = 0.25). The FEV1 and FVC values increased by 0.22 ± 0.24 L and 0.38 ± 0.30 L (*P* values < 0.001), respectively, while the mean FEV1/FVC ratio was reduced by 2.65% ± 6.06% (*p* value = 0.19). The average FEV1 and FVC percentages increased by 7.59% ± 8.92% and 10.53% ± 8.36%, respectively, postoperatively (Table 1). The absolute changes in FEV1 and FVC (*r* =.369, *P* =.038) values correlated significantly and positively with TWL percentage (Fig. 1). The FEV1 change was also correlated with EWL% (Fig. 2). The correlation between other respiratory function variables and TWL% was positive but non-significant (Table 2).

The mean distance that patients walked during 6MWT increased by 53.71 ± 75.17 m (*P* <.001) after SG (Table 1). The correlations between 6MWT changes and TWL% (*r* =.451, *p* =.01) was significant. However, EWL% was not correlated with 6MWT changes 12 months after SG (Table 2). The patient’s physical activity levels were significantly modified after SG. After one year, the weekly MET-minutes of cases increased by 1067.25 ± 1251.71 M/w. Before surgery, most of the cases were in the low-activity group (62.5%), while only 9.6% of patients remained low-active one year after surgery. The changes in physical activity levels were not correlated with TWL (*r* =.003, *p* =.98) or EWL (*r*=-.031, *p* =.86). The changes in LBM were negatively correlated with FEV1, FVC, and 6MWT changes (Table 2).

**Table 1:** The changes in anthropometric, body composition, respiratory function and physical activity variables before and after surgery

Variable	Before surgery	After surgery	Change	*P* value^1^
Weight (Kg)	111.47 ± 13.98	88.05 ± 12.44	-23.42 ± 11.78	< 0.001
BMI (Kg/m^2^)	43.86 ± 4.39	34.72 ± 4.76	-9.14 ± 4.49	< 0.001
Fat mass (Kg)	60.07 ± 9.52	43.88 ± 67.33	-16.18 ± 8.44	< 0.001
Lean body mass (Kg)	51.34 ± 6.45	44.06 ± 6.17	-7.26 ± 4.09	< 0.001
MIP (cmH_2_O)	62.11 ± 23.47	69.35 ± 19.86	7.24 ± 17.42	0.025
FEV1 (L)	2.50 ± 0.54	2.72 ± 0.58	0.22 ± 0.24	< 0.001
FEV1 (%)	87.12 ± 11.71	94.71 ± 9.92	7.59 ± 8.92	< 0.001
FVC (L)	3.02 ± 0.66	3.40 ± 0.77	0.38 ± 0.30	< 0.001
FVC (%)	87.18 ± 10.90	97.71 ± 9.58	10.53 ± 8.36	< 0.001
FEV1/FVC (%)	82.41 ± 5.44	79.75 ± 4.42	-2.65 ± 6.06	0.019
METs (M/w)	1238.75 ± 1631.72	2306.38 ± 1858.36	1067.25 ± 1251.71	<.001^2^
Activity level (n)				<.001^3^
Low	20 (62.5%)	3 (9.6%)	-	
Moderate	5 (15.6%)	16 (50.0%)	-	
High	7 (21.9%)	13 (40.6%)	-	
6MWTD (m)	439.75 ± 104.63	493.47 ± 90.72	53.71 ± 75.17	< 0.001

^1^based on the Paired sample T-test

^2^based on the Wilcoxon signed-rank test

^3^based on the Chi Square test

BMI = Body Mass index, MIP = Maximal Inspiratory Pressure, FEV1 = Expiratory Flow in the first second (FEV1), FVC = Forced Vital Capacity, METs = Metabolic Equivalents, 6MWT = 6-Minute Walk Test Distant

**Table 2:** The Pearson correlation coefficient *r*. Values between various variables and EWL% and TWL%

Variable	EWL%	TWL%	∆LBM	
∆FEV1	0.433^*^	0.470**	− 0.489**	
∆FVC	0.344	0.369^*^	− 0.370*	
∆FEV1/FVC	0.060	0.080	− 0.078	
∆MIP (CM H_2_O)	0.337	0.311	− 0.310	
∆METs (M/w)	− 0.031	0.003	− 0.063	
∆6MWT (m)	0.344	0.451^**^	− 0.391*	

^*^P value < 0.05

^**^P value < 0.01

EWL = Excess Weight Loss, TWL = Total Weight Loss, LBM = Lean Body Mass, MIP = Maximal Inspiratory Pressure, FEV1 = Expiratory Flow in the first second (FEV1), FVC = Forced Vital Capacity, METs = Metabolic Equivalents, 6MWT = 6-Minute Walk Test Distant

## Discussion

Previous studies observed impaired respiratory functions and altered lung volumes in patients with severe obesity. The accumulation of excessive adipose tissue around the thoracoabdominal area could reduce the chest wall compliance and expansion of the lung [5, 22]. Moreover, increased intra-abdominal pressure may limit the diaphragm’s movements and respiratory function [23]. Furthermore, systemic inflammation in obesity could cause congestion and restrict the size of the small airway by increasing pulmonary blood flow. For instance, some articles reported a negative association between the pro-inflammatory cytokine IL-6 and FVC [24, 25]. Several studies suggested that these alternations are reversible, and excessive weight loss could potentially modify respiratory function [22, 26, 27]. The primary finding of the current study was the significant modification in various anthropometric parameters, including BMI, Body weight, Fat mass, and Lean body mass, as well as respiratory function test (FEV1, FVC, and MIP), one year after SG in 32 individuals with obesity. Furthermore, the changes in FEV1 and FVC were significantly correlated in a positive direction with TWL, indicating the probable beneficial role of SG in modifying respiratory function. In 2020, Mihmanlı et al. investigated 135 patients with obesity and pathological findings in preoperative spirometry and observed a significant increase in FVC, FEV1, and FEV1/FVC ratio after SG. In contrast to our study, they did not find a significant correlation between pulmonary function tests (PFTs) and EWL. However, they observed a positive correlation between FEV1/FVC ratio changes and EWL [26]. The difference in results may be explained by the fact that they evaluated only cases with pathological spirometry findings. In a meta-analysis based on 23 studies, the authors reported that dynamic lung volume tests (FVC and FEV1) were improved moderately after various types of bariatric surgery [28]. An article with a longer follow-up also showed a significant improvement in lung functional tests 5 years after weight reduction surgery [29]. Furthermore, the study of 94 individuals with obesity who underwent RYGB surgery showed significant improvement in all PFTs even in a short follow-up (3 months) postoperatively. They also observed that weight change was significantly correlated to changes in FEV1 and FVC but not with FEV1/FVC ratio change [30]. Additionally, Borasio et al. stated a significant increase in FEV1 and FVC values after SG in 47 cases with severe obesity. Still, they did not indicate a significant change in the FEV1/FVC ratio [5]. These findings were in agreement with our study. Although our study showed significant improvement in FEV1 and FVC percentages, the FEV1/FVC ratio was not significantly reduced. The reduced FEV1/FVC ratio usually represents the development or exacerbation of airflow obstruction. These findings revealed the beneficial role of weight loss surgery in improving respiratory function primarily due to the modification of restrictive lung mechanisms. However, our cases did not have any preexisting obstructive lung disease, and larger and more diverse studies are needed to confirm the observed improvement.

On the other hand, Souza et al. indicated that only the FVC test was significantly increased after Roux-en-Y gastric bypass surgery, and improvements in FEV1, FEV1/FVC ratio, and expiratory reserve volume were non-significant at one year after surgery [31]. Previous studies hypothesize that respiratory muscle strength may be reduced in patients with obesity for various reasons. They suggest lung expansion is restricted in individuals with obesity due to a higher elastic load on the thoracic cage, making inspiration more difficult for inspiratory muscles. They also state that respiratory muscles become less strong and effective in cases with obesity because of fat deposition in the thoracoabdominal area. In the current case series, we observed reduced mean MIP (62.11 cmH_2_O) in our patients before surgery compared to the normal predicted values reported in previous studies and meta-analysis [8, 32–34]. Weiner et al. reported a significant increase of 21 cm H_2_O in mean MIP six months after gastroplasty [8]. Later, in 2004, a study observed a significant improvement in MIP values one year after weight-loss surgery [9]. De Campos et al. indicated that although the preoperative mean MIP of cases was within the normal predicted range, patients experienced a 10 cm H_2_O increase in MIP after BS [35]. We also observed a significant increase of 7.24 cm H_2_O in the MIP of our patients one year after SG. Despite the significant improvement, the mean MIP of cases remained lower compared to the healthy adults of the same age and gender [34], revealing that surgery could not fully reverse the muscle weakness observed among the subjects. Previous studies demonstrated that a relatively small increase in the MIP could also lead to better lung expansion [8, 35]. In conclusion, our findings and literature review suggested that bariatric surgery could benefit the lung function and respiratory muscle strength of patients with obesity.

Physical function is another essential aspect of the life of patients with obesity, which has a profound effect on both the mental and physical health of patients. A study by Tompkins et al. investigating 25 cases with obesity who underwent gastric bypass surgery indicated a gradual increase in 6MWT distance 3 and 6 months after BS [36]. Multiple studies observed the same findings over time [11, 37–40]. The 6MWT distance increase ranged from 8.8 to 33.3% after metabolic surgery in various studies [41]. The current analysis also demonstrated a 12.2% increase in walked distance, equivalent to an improvement of 53.71 ± 75.17 m (*p* <.001) in patients’ 6MWT one year after SG. This change is clinically meaningful, as previous research suggests that slight improvements in 6MWT distance can significantly enhance functional capacity and quality of life in patients with obesity. Studies have shown that an increase of 30 to 50 m in 6MWT distance has been associated with reduced dyspnea, improved endurance, and greater ability to perform daily activities [15, 37]. Improved physical function can be partially explained by reduced dyspnea resulting from weight loss and enhanced respiratory function, which together encourage patients to engage more in PA and improve their endurance. Previous studies supported the observed correlation between weight loss and an increase in PA in a positive direction [42]. However, the causality between these two parameters is still unclear; it’s difficult to determine whether patients exercised more due to greater weight loss or experienced greater weight loss because they exercised more. Modified muscle, cardio, and respiratory function probably helped patients experience a more active life post-operatively [42, 43]. Our study revealed a significant negative correlation between changes in LBM and 6MWT distance, indicating that patients with smaller reductions in LBM tended to achieve greater improvements in 6MWT performance. This finding suggests that preserving LBM may play a role in enhancing functional capacity following surgery.

The GPAQ questionnaire was also employed to evaluate the changes in physical activity of patients with obesity after SG. The validity and adaptability of this questionnaire were previously proven [44]. In 2017, a study showed a significant increase in the physical activity of 26 individuals with obesity six months after SG by 1362 M/w in mean MET-minutes [45]. Furthermore, in 2024, Bullo et al. observed a significant modification of 928.7 M/w in the mean total weekly activity of 60 women with obesity six months after SG [46]. Our findings regarding the changes in physical activity were consistent with previous studies. In the current study, patients experienced a mean increase of 1067.25 ± 1251.71 M/w in their total weekly activity 12 months after SG. However, our analysis did not indicate a correlation between changes in MET minutes and weight loss. This finding may contribute to the follow-up time since Boddu’s study did not observe this correlation after six months, but they observed it 18 months after surgery [21]. Furthermore, various physical and psychosocial factors, including adherence to dietary recommendations, hormonal adaptations, BMR changes, and psychological disorders, determined weight loss outcomes after surgery [47, 48]. During the early postoperative period, weight loss is predominantly caused by calorie restrictions and hormonal changes induced by surgery rather than PA [48]. Therefore, the correlation between PA and weight loss may observed in an extended period of follow-up.

In summary, our findings reveal that the benefits of LSG extend beyond weight loss, with significant improvements in respiratory function, physical capacity, and activity levels observed in our cohort. However, to maximize these benefits, we recommend that clinicians incorporate structured exercise programs, particularly those emphasizing resistance training, into postoperative care to prevent the excessive loss of LBM. Additionally, long-term monitoring of patients is essential to evaluate changes in body composition, activity levels, and adherence to postoperative recommendations.

This study has multiple limitations that require attention in future research in the metabolic surgery field. Body composition and respiratory function before or after BS may vary between men and women [49]. However, studying the differences between genders was impractical since only four male patients were included in the current article. Moreover, physical activity levels were evaluated using the GPAQ questionnaire. Although this tool has been shown to have relatively good reliability, its efficacy may be lower than objective methods [50]. Additionally, a relatively small sample size of the current study could potentially limit the clinical applicability of findings. Another limitation was using a body impedance analyzer to assess body composition instead of dual-energy X-ray absorptiometry (DXA), which is considered the gold standard for this measurement. Further studies with larger sample sizes and extended follow-up durations are required to validate our findings.

## Data Availability

The datasets utilized and examined in the present study can be obtained from the corresponding author upon making a reasonable request.
